# Supplementary material for: Asymmetric apical domain states of mitochondrial Hsp60 coordinate substrate engagement and chaperonin assembly
Source: Nat Struct Mol Biol. 2024 Jul 1;31(12):1848–58. doi: 10.1038/s41594-024-01352-0 (PMC11638070; doi:10.1038/s41594-024-01352-0)
Supplement: Supplementary file 1 — Reporting Summary [file 41594_2024_1352_MOESM1_ESM.pdf]

Corresponding author(s): Daniel R. Southworth, Jason E. Gestwicki

Last updated by author(s): Apr 28, 2024

Reporting Summary

Nature Portfolio wishes to improve the reproducibility of the work that we publish. This form provides structure for consistency and transparency in reporting. For further information on Nature Portfolio policies, see our Editorial Policies and the Editorial Policy Checklist.

Statistics

For all statistical analyses, confirm that the following items are present in the figure legend, table legend, main text, or Methods section.

n/a	Confirmed	
☐	☒	The exact sample size (*n*) for each experimental group/condition, given as a discrete number and unit of measurement
☐	☒	A statement on whether measurements were taken from distinct samples or whether the same sample was measured repeatedly
☐	☒	The statistical test(s) used AND whether they are one- or two-sided<br>*Only common tests should be described solely by name; describe more complex techniques in the Methods section.*
☒	☐	A description of all covariates tested
☐	☒	A description of any assumptions or corrections, such as tests of normality and adjustment for multiple comparisons
☐	☒	A full description of the statistical parameters including central tendency (e.g. means) or other basic estimates (e.g. regression coefficient) AND variation (e.g. standard deviation) or associated estimates of uncertainty (e.g. confidence intervals)
☐	☒	For null hypothesis testing, the test statistic (e.g. *F*, *t*, *r*) with confidence intervals, effect sizes, degrees of freedom and *P* value noted<br>*Give P values as exact values whenever suitable.*
☒	☐	For Bayesian analysis, information on the choice of priors and Markov chain Monte Carlo settings
☒	☐	For hierarchical and complex designs, identification of the appropriate level for tests and full reporting of outcomes
☒	☐	Estimates of effect sizes (e.g. Cohen's *d*, Pearson's *r*), indicating how they were calculated

Our web collection on statistics for biologists contains articles on many of the points above.

Software and code

Policy information about availability of computer code

Data collection	Electron microscopy data were collected using SerialEM 4.1.0, analytical chromatography data were collected using UNICORN 7, SEC-MALS data were collected using ASTRA V, and ATPase and mtMDH refolding data were collected using SoftMax Pro v7.
Data analysis	Data were analyzed using MotionCor2 v1.6.4, cryoSPARC v3.3, RELION v3.1, Coot v0.8.9.2, ISOLDE v1.3, Phenix v1.20.1, UCSF Chimera v1.16, UCSF ChimeraX v1.3, MUSCLE v5, MView v1.67, Adobe Illustrator 2023, and GraphPad Prism v9.3.1.

For manuscripts utilizing custom algorithms or software that are central to the research but not yet described in published literature, software must be made available to editors and reviewers. We strongly encourage code deposition in a community repository (e.g. GitHub). See the Nature Portfolio guidelines for submitting code & software for further information.

Data

Policy information about availability of data

All manuscripts must include a data availability statement. This statement should provide the following information, where applicable:

- Accession codes, unique identifiers, or web links for publicly available datasets
- A description of any restrictions on data availability
- For clinical datasets or third party data, please ensure that the statement adheres to our policy

Cryo-EM densities have been deposited at the Electron Microscopy Data Bank under accession codes EMD: 29813 (mtHsp60apo consensus), EMD: 29814 (mtHsp60apo focus), EMD: 29815 (mtHsp60ATP consensus), EMD: 29816 (mtHsp60ATP focus), EMD: 29817 (mtHsp60ATP-mtHsp10 consensus), and EMD: 29818

(mtHsp60ATP-mtHsp10 focus). Atomic coordinates have been deposited at the Protein Data Bank under accession codes PDB: 8G7J (mtHsp60apo consensus), PDB: 8G7K (mtHsp60apo focus), PDB: 8G7L (mtHsp60ATP consensus), PDB: 8G7M (mtHsp60ATP focus), PDB: 8G7N (mtHsp60ATP-mtHsp10 consensus), and PDB: 8G7O (mtHsp60ATP-mtHsp10 focus). Accession codes for additional models referenced in this study are PDB 7AZP (mtHsp60 apo), PDB 6MRC (mtHsp60ADP-mtHsp10), PDB 4KI8 (GroEL R-ADP), PDB 4AAR (GroEL Rs2), PDB 1AON (GroELADP-GroES), PDB 1SVT (GroELADP-AlFx-GroES), and PDB 1KP8 (GroELATP). An uncropped image for Extended Data Fig. 1f and data used for all biochemical experiments are provided as source data online.

Human research participants

Policy information about studies involving human research participants and Sex and Gender in Research.

Reporting on sex and gender	N/A
Population characteristics	N/A
Recruitment	N/A
Ethics oversight	N/A

Note that full information on the approval of the study protocol must also be provided in the manuscript.

Field-specific reporting

Please select the one below that is the best fit for your research. If you are not sure, read the appropriate sections before making your selection.

☒ Life sciences ☐ Behavioural & social sciences ☐ Ecological, evolutionary & environmental sciences

For a reference copy of the document with all sections, see nature.com/documents/nr-reporting-summary-flat.pdf

Life sciences study design

All studies must disclose on these points even when the disclosure is negative.

Sample size	Cryo-EM images were collected for each sample until there was a reasonable expectation of performing reconstructions at the desired resolution (~3 Angstrom, sufficient for atomic model building). In all cases the desired resolutions were reached, indicating that the sample sizes were sufficient. Biochemical experiments were performed in biological duplicate or triplicate and technical triplicate (where applicable), in keeping with standard practice in the field. Replication attempts yielded similar results in all cases, indicating that the sample sizes used were sufficient to capture experimental variability.
Data exclusions	Micrographs were excluded based on poor maximum estimated resolution. Particles were excluded during 2D and 3D classification in cryoSPARC and RELION based on assignment to low-resolution or otherwise artifactual classes. One biological replicate of the mtMDH folded control in the mtMDH refolding experiments showed no activity (likely due to technical error); data from this replicate were excluded from analysis.
Replication	Biochemical experiments were performed in biological duplicate or triplicate and and technical triplicate (where applicable). All replication attempts were successful, with the exception of the biological replicate of the mtMDH folded control in the mtMDH refolding experiments discussed above. Cryo-EM data were randomly divided into two halves that were independently refined.
Randomization	Cryo-EM data was randomly assigned to two half sets during refinement. Resolution estimates are based on comparisons of reconstructions from these half sets. Biochemical experiments were not randomized as no subjective assessment of data was required.
Blinding	Investigators were not blinded in any cryo-EM or biochemical data collection or analysis as experimental conditions needed to be known to prepare samples, acquire data, and analyze results; furthermore, no subjective assessment of data was required.

Reporting for specific materials, systems and methods

We require information from authors about some types of materials, experimental systems and methods used in many studies. Here, indicate whether each material, system or method listed is relevant to your study. If you are not sure if a list item applies to your research, read the appropriate section before selecting a response.

Materials & experimental systems

n/a	Involved in the study
☒	☐ Antibodies
☒	☐ Eukaryotic cell lines
☒	☐ Palaeontology and archaeology
☒	☐ Animals and other organisms
☒	☐ Clinical data
☒	☐ Dual use research of concern

Methods

n/a	Involved in the study
☒	☐ ChIP-seq
☒	☐ Flow cytometry
☒	☐ MRI-based neuroimaging
