# Supplementary figures and images for: Asymmetric apical domain states of mitochondrial Hsp60 coordinate substrate engagement and chaperonin assembly
Source: Nat Struct Mol Biol. 2024 Jul 1;31(12):1848–58. doi: 10.1038/s41594-024-01352-0 (PMC11638070; doi:10.1038/s41594-024-01352-0)

Source Data for Extended Data Fig. 1f | Uncropped gel.

1  $\mu\text{M}$   
5  $\mu\text{M}$   
(shown in figure)  
10  $\mu\text{M}$

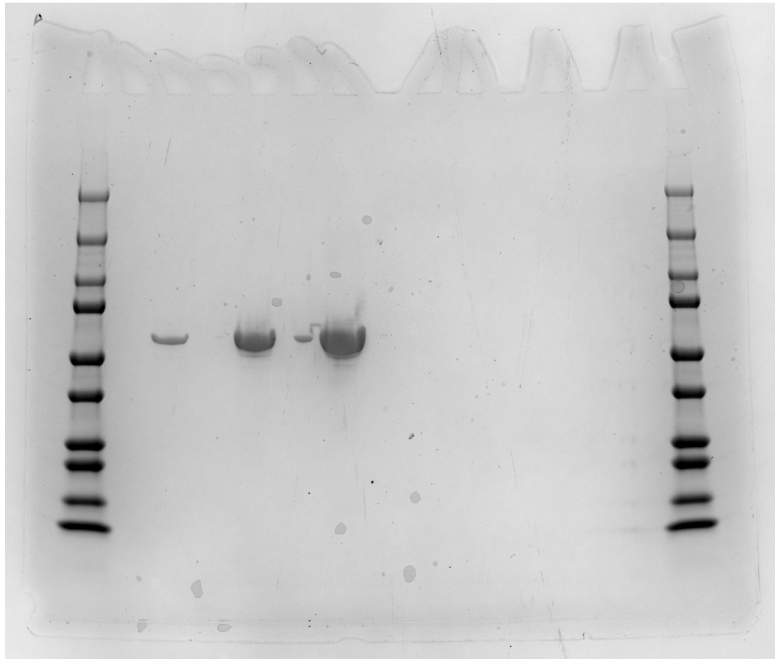

Supplement: Supplementary file 8 — Uncropped gel. [file 41594_2024_1352_MOESM8_ESM.pdf]
